# Evidence of a Role for the TRPC Subfamily in Mediating Oxidative Stress in Parkinson’s Disease

**DOI:** 10.3389/fphys.2020.00332

**Published:** 2020-05-08

**Authors:** Daniele Maria-Ferreira, Natalia Mulinari Turin de Oliveira, Liziane Cristine Malaquias da Silva, Elizabeth Soares Fernandes

**Affiliations:** ^1^Faculdades Pequeno Príncipe, Programa de Pós-graduação em Biotecnologia Aplicada à Saúde da Criança e do Adolescente, Curitiba, Brazil; ^2^Instituto de Pesquisa Pelé Pequeno Príncipe, Curitiba, Brazil

**Keywords:** TRPC channels, Parkinson’s disease, oxidative stress, dopamine release, neuronal apoptosis

## Abstract

Parkinson’s disease (PD) represents one of the most common multifactorial neurodegenerative disorders affecting the elderly population. It is associated with the aggregation of α-synuclein protein and the loss of dopaminergic neurons in the substantia nigra pars compacta of the brain. The disease is mainly represented by motor symptoms, such as resting tremors, postural instability, rigidity, and bradykinesia, that develop slowly over time. Parkinson’s disease can also manifest as disturbances in non-motor functions. Although the pathology of PD has not yet been fully understood, it has been suggested that the disruption of the cellular redox status may contribute to cellular oxidative stress and, thus, to cell death. The generation of reactive oxygen species and reactive nitrogen intermediates, as well as the dysfunction of dopamine metabolism, play important roles in the degeneration of dopaminergic neurons. In this context, the transient receptor potential channel canonical (TRPC) sub-family plays an important role in neuronal degeneration. Additionally, PD gene products, including DJ-1, SNCA, UCH-L1, PINK-1, and Parkin, also interfere with mitochondrial function leading to reactive oxygen species production and dopaminergic neuronal vulnerability to oxidative stress. Herein, we discuss the interplay between these various biochemical and molecular events that ultimately lead to dopaminergic signaling disruption, highlighting the recently identified roles of TRPC in PD.

## Introduction

Neurological disorders continue to increase in tandem with longer lifespans in populations, with aging remaining the biggest risk factor for developing neurodegenerative diseases. Parkinson’s disease (PD) is one of the most common multifactorial neurodegenerative disorders. Indeed, it affects approximately 2% of the elderly population and 4% of individuals aged over 80 years (Berman and Nichols, 2019).

Disease onset usually occurs at the age of 65–70 years (Marino et al., 2019). However, its pathological changes can be observed as early as 20 years prior to the appearance of motor symptoms and include unspecific signs such as fatigue, hyposmia, and constipation (Hawkes et al., 2010). Motor symptoms develop slowly over time and are the main clinical characteristics of PD. These include dysfunctions of the somatomotor system such as resting tremors, rigidity, bradykinesia, and postural instability (Schapira et al., 2017). In turn, there is a progressive physical limitation, in addition to impairments in non-motor functions such as neuropsychiatric (sleep disorders, depression, and dementia) and autonomic symptoms (bladder and gastrointestinal alterations) (Sakakibara et al., 2012; Fasano et al., 2015).

The pathogenesis of PD is not completely understood. However, different studies have contributed to the dissection and determination of some of the mechanisms involved in its establishment and progression. Classically, the progressive neurodegeneration in PD is associated with the aggregation of α-synuclein, a small lipid-binding protein, into structures called Lewy bodies in the substantia nigra pars compacta (SNpc).

Accumulation of dopamine (DA) and DA products has also been pointed as a potential mechanism involved in neuronal death (Mullin and Schapira, 2015). Indeed, the neurotoxic effects of the endogenous DA derivative *N*-methyl-(*R*)-salsolinol (NMSAL) (Naoia et al., 2002) was shown to induce oxidative stress and decrease the levels of reduced glutathione (GSH) in dopaminergic SH-SY5Y cells (Wanpen et al., 2004). The progressive loss of DA neurons leads to a subsequent reduction of DA levels. All these alterations contribute to an abnormal neuronal functioning, and thus, to motor deficiency and worsening of the quality of life of patients at advanced stages of PD (Magrinelli et al., 2016).

For instance, many studies have provided substantial evidence of the role of neuroinflammation (Tansey and Goldberg, 2010), mitochondrial dysfunction (Park et al., 2018, 2019), and oxidative and nitrosative stresses in PD (Puspita et al., 2017). In this context, disruption of neuronal calcium ion (Ca^2+^) homeostasis in the central nervous system plays a critical role in the cascade of events that culminates in the degeneration of dopaminergic neurons (Zaichick et al., 2017). Also, a correlation between reactive oxygen species (ROS) production and Ca^2+^ channel activation has already been explored (Görlach et al., 2015).

Recent studies have focused in the identification of a link between Ca^2+^-mediated signaling and neuroinflammation (Sama and Norris, 2013). It observed an association between neurodegeneration, mitochondrial dysfunction, and, oxidative and nitrosative stresses (Celsia et al., 2009). This evidence points to a role for transient receptor potential channels (TRP) in PD (Takahashi and Mori, 2011).

First discovered in *Drosophila melanogaster* as key molecules in phototransduction, the TRP channels comprise a family of non-selective cation channels that are widely expressed on mammalian cells, including neurons and different types of non-neuronal cells. They are distributed in six different subfamilies: ankyrin (TRPA1), canonical (TRPC1-7), melastatin (TRPM1-8), mucolipin (TRPML1-3), polycystin (TRPP1-3), and vanilloid (TRPV1-6). Their broad tissue expression confers them the ability to influence different pathologies and physiological states. In this context, it is now known that these channels participate in the development and maintenance of inflammation and pain, are important sensors of molecules such as lipids and ROS, and are involved in thermoregulation, tissue remodeling, and neuronal plasticity, among other responses.

## Oxidative and Nitrosative Stresses in Parkinson’s Disease

**FIGURE 1:**
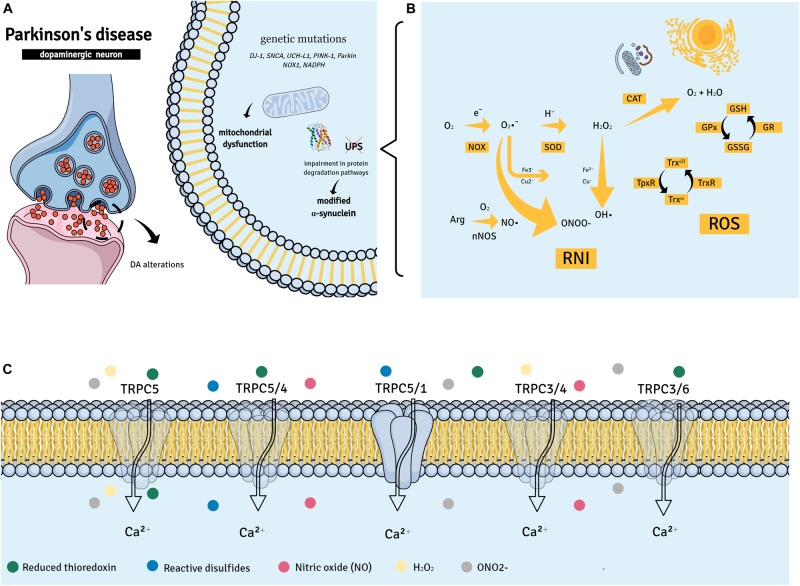
Parkinson’s disease (PD) suggested pathways. **(A,B)** PD has been associated with the aggregation of α-synuclein into *Lewy* bodies in dopaminergic neurons of the substantia nigra pars compacta. Other factors such as gene mutations (DJ-1, SNCA, UCH-L1, PINK-1, and Parkin) may contribute to mitochondrial dysfunction and neuronal death in PD. The accumulation of dopamine (DA) and its products in DA neurons may also be a causative factor of neuronal death. This may lead to mitochondrial dysfunction, changes in protein degradation [by impairing the ubiquitin-proteasome system (UPS) function], and increased generation of reactive oxygen species (ROS) and reactive nitrogen intermediates (RNIs). **(C)** Members of the transient receptor potential canonical (TRPC) subfamily of non-selective Ca^2+^ channels are able to recognize ROS and RNIs and have been implicated in neuronal survival; in fact, different oxidative/nitrosative stress products can directly activate TRPC complexes.

Reactive oxygen species and reactive nitrogen intermediates (RNIs) are natural byproducts necessary for cellular homeostasis (Liguori et al., 2018) (Figure 1). ROS are formed during metabolic redox reactions and include hydrogen peroxide (H_2_O_2_), singlet oxygen (^1^O_2_), hydroxyl (∙OH), and superoxide (O_2_∙−) radicals (Sies et al., 2017). RNIs are produced in neuronal cells from arginine by the neuronal nitric oxide synthase (nNOS) and include nitric oxide (NO∙), nitrite (NO_2_), and *S*-nitrosothiols and peroxynitrite (OONO−) (Adams et al., 2015).

Excessive ROS and RNI formation during oxidative and nitrosative stresses results in a variety of detrimental effects in the cell, thus, contributing to organelle and membrane structural damages and cellular apoptosis (Guo et al., 2018). This cytotoxic environment has been recognized as a common underlying phenomenon in the dopaminergic neurodegenerative process (Dias et al., 2013). Indeed, an irregular oxidation of macromolecules, such as lipids, proteins, and nucleic acids, was observed in the brain tissues of PD patients (Bosco et al., 2006; Nakabeppu et al., 2007). Also, higher levels of the oxidative stress markers 8-OhdG (8-Oxo-2′-deoxyguanosine) and malondialdehyde, in addition to NO_2_, were detected in the peripheral blood of PD patients in comparison with healthy individuals (Wei et al., 2018). The same patients presented systemic down-regulation of the antioxidant proteins glutathione and catalase (CAT).

In addition, major genetic insights indicate that specific mutations in a series of primary genes that are responsible for PD-related synucleopathy and the regulation of mitochondrial and ROS equilibrium can disrupt cellular homeostasis (Cacabelos, 2017). For instance, an elevated expression of the wα-synuclein protein and oxidative stress genes [*HSPB1*, Heat Shock Protein Family B (Small) Member 1; *NOX1*, NADPH oxidase 1; and *MAOB*, Monoamine oxidase B] was observed in induced pluripotent stem cell (iPSC)-derived dopaminergic neurons (Nguyen et al., 2011). Similarly, *iPSC* midbrain dopaminergic neurons from patients with PTEN-induced putative kinase 1 (*PINK1*) or *Parkin* mutations presented abnormal mitochondria (Chung et al., 2016) (Figure 1).

Accordingly, evidence suggests that in PD, the mitochondrion represents the primary source of ROS, contributing to intracellular oxidative stress and therefore, to the vulnerability of dopaminergic neurons to apoptosis (Beal, 2005). Moreover, knockout mice for Dynamin-1-like protein (*Drp1*), a guanosine triphosphate (GTP)ase that regulates mitochondrial fission, exhibited degeneration of nigrostriatal dopaminergic neurons (Berthet et al., 2014). This response was associated with a reduced mitochondrial mass in axons, which was associated with impaired mitochondrial dynamics denoted by the loss of coordination of mitochondrial movements.

Additionally, disruption of respiratory chain complexes, especially the mitochondrial complex I (NADH-quinone oxidoreductase), was implicated in the enhanced production of ROS in PD (Ryan et al., 2015). Human studies also indicated that the dysfunction of this specific complex occurs in the SNpc of PD patients (Schapira et al., 1990). Of note, mitochondrial integrity in SNpc neurons was found to be dependent on Parkin expression (Park et al., 2006; Stichel et al., 2007).

In regard to RNIs, the excessive or inappropriate generation of NO and O_2_∙−-derived reactive species, plays a critical role in mediating the neurotoxicity associated with mitochondrial damage (Kaludercic and Giorgio, 2016). The reaction between NO and O_2_∙− represents an important source of OONO−, a highly reactive molecule for a broad range of chemical targets that potently inhibits mitochondrial proteins. OONO− overproduction was found to enhance the levels of oxidized lipids and DNA in the dopaminergic neurons of PD patients (Ebadi and Sharma, 2003). Depletion of antioxidant defenses, including GSH, was also observed in the same samples (Franco and Cidlowski, 2009). Interestingly, nNOS- and inducible NO synthase (iNOS)-dependent NO levels were increased in the SNpc of PD patients (Hancock et al., 2008). Also, high levels of NO and OONO− correlated with a worse prognosis in PD (Kouti et al., 2013), corroborating the hypothesis that both RNI and ROS generation may strongly contribute to neurodegeneration in PD.

Antioxidant proteins such as superoxide dismutase (SOD), CAT, glutathione peroxidase (GPx), and GSH counteract excessive ROS production. Therefore, reductions in their activities and/or expression may favor lipid peroxidation or promote neuronal excitotoxicity with subsequent protein modifications and eventual neuronal death (Deponte, 2013; Patlevič et al., 2016). Interestingly, evident differences were found in the levels of GSH of post-mortem brain samples of PD patients in comparison with other brain regions (Perry et al., 1982; Sian et al., 1994). Also, animal studies revealed that down-regulation of GSH synthesis results in a progressive degeneration of nigrostriatal dopaminergic neurons (Garrido et al., 2011).

By using agonists and antagonists, knockout mice and cells, and a diverse range of molecular biology techniques, several roles have been suggested for the TRPC subfamily. These include their importance as sensors of molecules involved in oxidative and nitrosative stresses (Figure 1) known to influence neuronal survival and function (Chen et al., 2009; Delgado-Camprubi et al., 2017).

## Transient Receptor Potential Channels and the Canonical Subfamily

In humans, the TRPC subfamily is formed by six channels (TRPC1 and TRPC3-7), which are considered the mammalian TRPs most closely related to those of *D. melanogaster*. TRPC channels are formed by four subunits and each subunit has six transmembrane domains and a pore region between the fifth and the sixth transmembrane domain (Feng, 2017).

TRPCs assemble into tetramers to form functional channels. Each monomer consists of a transmembrane domain and a cytosolic domain (Li et al., 2019). The cytosolic domain contains the N- and C-terminal subdomains. The N-terminal is composed of four ankyrin repeats and linker helices, whilst the C-terminal is formed by a connecting helix and a coiled-coil domain (Li et al., 2019). All TRPC channels contain the calmodulin and inositol trisphosphate (IP_3_) receptor-binding motif, which is able to interact with phosphoinositides, inositol polyphosphates, Gαi/o proteins, and SEC14 domain and spectrin repeat-containing protein 1 (SESTD1), a Ca^2+^-dependent phospholipid/cytoskeleton-binding protein (Wang et al., 2020). These different interacting pathways may influence TRPC functions.

Distributed in two subgroups, diacylglycerol (DAG)-activated (TRPC3/6/7) and non-DAG-activated receptors (TRPC1/4/5), TRPC channels can form homo- and heterotetramers (Strübing et al., 2001; Zagranichnaya et al., 2005; Poteser et al., 2006; Woo et al., 2014; Myeong et al., 2016; Bröker-Lai et al., 2017; Sunggip et al., 2018; Ko et al., 2019). Their assembly in these complexes may vary with their expression sites and functions. Additionally, members of the TRPC subfamily, such as TRPC1, can also form heterotetramers with channels of other TRP subfamilies, including TRPV4 and TRPP2 (Kobori et al., 2009; Greenberg et al., 2017). Despite the advances in elucidating the structure and assembly of TRPCs, the definite functions of their homo- and heterotetramers remain unclear and represent a whole new avenue of knowledge to be pursued.

So far, different roles have been identified for TRPC channels including in cardiovascular, lung, kidney and neurological diseases, inflammation, and cancer, among others. Of importance to our review, TRPCs are involved in neurotransmission, neural development, excitotoxicity, and neurodegeneration (Wang et al., 2020). Interestingly, TRPC channels, especially TRPC1, have topped the list of molecules involved in store-operated Ca^2+^ entry. However, it is now well-established that their importance goes beyond the endoplasmic reticulum Ca^2+^ store (Wang et al., 2020). Herein, we will focus on the importance of TRPC channels as oxidative and nitrosative sensors in PD.

In regard to oxidative stress, TRPC5 is perhaps the most well investigated member of the TRPC subfamily. It can be activated by both oxidant and antioxidant molecules such as H_2_O_2_ and reduced thioredoxin, respectively (Yoshida et al., 2006; Xu et al., 2008; Naylor et al., 2011). TRPC5 can be also activated by NO and reactive disulfides (Yoshida et al., 2006; Maddox et al., 2018). However, TRPC5 sensitivity to NO has been argued by other studies (Xu et al., 2008; Wong et al., 2010), indicating this response may vary with cell type, generated NO concentrations, and other experimental conditions. Interestingly, TRPC5/TRPC4 complexes were found to be involved in the regulation of Ca^2+^-dependent production of NO by endothelial cells (Yoshida et al., 2006). TRPC5-dependent NO generation via endothelial NOS (eNOS) activation was later confirmed (Sunggip et al., 2018).

Another interesting finding is the ability of oxidant products such as OONO− to up-regulate both the mRNA and protein expressions of TRPC6 and TRPC3 in monocytes. Of note, OONO−-induced Ca^2+^ influx in these cells is reversed by the TRPC channel blocker 2-APB (Wuensch et al., 2010). Additionally, TRPC3/TRPC4 assembly forms redox-sensitive complexes on endothelial cells (Poteser et al., 2006). Adding another layer of complexity to TRPC roles in oxidative/nitrosative stresses, it is important to highlight that these channels do not only form complexes but are also able to down-regulate each other’s’ responses. Indeed, TRPC3/TRPC6-mediated Ca^2+^ influx can be down-regulated by activation of the TRPC5-NO axis (Sunggip et al., 2018).

Evidence also indicates that TRPC1 negatively regulates TRPC5-mediated Ca^2+^ influx in striatal neurons undergoing oxidative stress (Hong et al., 2015). Interestingly, TRPC1/TRPC5 complexes have been shown to mediate the protective effects of reduced thioredoxin in inflammation, therefore acting as a target for this antioxidant molecule (Xu et al., 2008).

Importantly, TRPCs are highly expressed in various regions of the brain in which they play different roles (Table 1). Thus, due to their ability to sense and modulate oxidative/nitrosative stress responses, they should be considered as potential mediators of neuroinflammation. Therefore, the importance of TRPC channels in PD will now be discussed.

**TABLE 1:** Neuronal expression and functions of TRPC channels.

**Receptor**	**Animal species/strains/cell lines**	**Expression site**	**Possible roles/effects following activation**	**References**
**TRPC1**	Sprague-Dawley rats	Telencephalon	Renewal of neural stem cells	Fiorio Pla et al., 2005;
	Wistar rats	cerebellum, and midbrain cortical pyramidal and SNpc neurons	Modulation of neuronal firing somato-dendritic release of dopamine following activation of mGluR and synaptic plasticity	Martorana et al., 2006; Valero et al., 2015; Martinez-Galan et al., 2018
	C57BL/6J mice	Hippocampal neural progenitor cells and neurons	Mediation of store-operated Ca^2+^ entry and neuronal cell differentiation and mediation of glutamate-induced cell death	Narayanan et al., 2008; Li et al., 2012
	SH-SY5Y cells and TRPC1 wild type and knockout mice (C57BL/6J background)	Neuroblastoma cells and mouse DA neurons from SNpc	Increased cell survival	Selvaraj et al., 2012
	Human	Brain cortical lesions from epilepsy patients and healthy tissues,	Mediation of astrocyte-induced epilepsy	Zang et al., 2015
	Cell line	D54 human glioma cells, H19-7 hippocampal neurons, PC12 cells	Store-operated Ca^2+^ entry and activation of Cl^–^ channels, differentiation of hippocampal neuronal cells, stimulation of neurite outgrowth and down-regulation of TRPC5-mediated responses	Wu et al., 2004; Heo et al., 2012; Cuddapah et al., 2013
**TRPC3**	Sprague-Dawley rats	Cerebellum, striatal cholinergic interneurons, striatal cholinergic interneurons, cortical neurons	Increased neuronal survival, modulation of the tonic activity of striatal cholinergic interneurons following activation of mGluR1/5, neuronal depolarization via interaction with dopamine receptors, mediation of low calcium and magnesium-induced depolarization, epileptiform activity, and redox-signaling	Berg et al., 2007; Jia et al., 2007; Roedding et al., 2013; Xie and Zhou, 2014; Zhou and Roper, 2014
	Wistar rats	Hippocampus	Integrity of the neuronal morphology, synaptic plasticity and cognition	Qin et al., 2015
	Balb/c	Prefrontal cortex	Depression-like behavior	Buran et al., 2017
	Wild type and *Mwk* mice	Cerebellum	Regulation of Purkinje cell development and survival, and synaptic plasticity	Becker et al., 2009; Dulneva et al., 2015
	C57Bl6J/SJL, and TRPC3 wild type and knockout (Sv129 background)	Hippocampus	Decrease in neuronal excitability, and early-onset memory deficits	Neuner et al., 2015
	Human	Cerebellar Purkinje neurons	Downstream signaling to mGluR activation; contribution of the TRPC3c isoform to focal ischaemic brain injury	Cederholm et al., 2019
	Cell line	H19-7 hippocampal neurons	Differentiation of hippocampal neuronal cells via store-operated calcium entry	Wu et al., 2004
**TRPC4**	TRPC4 wild type and knockout rats	Dopamine neurons	Dopaminergic activity and cocaine addition	Klipec et al., 2016
	C57BL/6 mice	Hippocampus, cortex, olfactory bulb, lateral septum, coronal brain slices, and prefrontal cortex	Neuronal development, anxiety, and depression	Zechel et al., 2007; Yang et al., 2015; Just et al., 2018
	Gonadotropin-releasing hormone (GnRH) transgenic mice	GnRH neurons from the pre-optic area	Sustained excitation of GnRH neurons and gonadotropin release	Zhang et al., 2013
	TRPC4 wild type and knockout mice (mixed background)	Amygdala, hippocampus, lateral septum, and hippocampus	Innate fear responses, downstream signaling to mGluR activation, seizure-induced excitotoxicity and neurodegeneration	Phelan et al., 2012; Riccio et al., 2014
	BL/6 P0 mice	Hippocampal neurons	Inhibition of neurite outgrowth	Jeon et al., 2013
	Human	Brain cortical lesions from epilepsy patients and healthy tissues	Seizure events	Wang et al., 2017
	Cell line	PC12 cells	Exocytosis in neuroendocrine cells	Obukhov and Nowycky, 2002
**TRPC5**	Sprague-Dawley rats	Pyramidal and hippocampal neurons	Seizure events, inhibition of dendritic development	Tai et al., 2011; He et al., 2012
	C57BL/6 mice	Coronal brain slices, cerebellar granular neurons, hippocampus, prefrontal cortex and retinal ganglion cells	Anxiety and depression, neuronal regeneration, retinal ganglion cell death	Yang et al., 2015; Wu et al., 2016; Just et al., 2018; Oda et al., 2019
	TRPC5 wild type and knockout mice (129/SvImJ background)	Cortical neurons	Oxidative stress-induced neuronal cell death	Park et al., 2019
	YAC128 mutant Huntington’s disease transgenic mice	Striatal cells	Oxidative stress-induced neuronal damage	Hong et al., 2015
	TRPC5 wild type and knockout mice (C57BL/6 and 129/SvImJ mixed background)	Hippocampus and amygdala	Fear-related responses	Riccio et al., 2009
	Human	Brain cortical lesions from epilepsy patients and healthy tissues	Seizure events	Xu et al., 2015
	Cell line	E18 hippocampal neurons, PC12 cells, NG108-15 neuroblastoma/glioma hybrid cells	Axon formation, neuronal development and plasticity, growth cone morphology and motility, neuronal regeneration	Greka et al., 2003; Wu et al., 2007; Davare et al., 2009; Wu et al., 2016
**TRPC6**	Sprague-Dawley rats	Cerebellum and substantia nigra	Neuronal survival, downstream signaling to mGluR activation	Giampà et al., 2007; Jia et al., 2007
	C57BL/6J mice TRPC6 wild type and over-expressing mice Cell line	Hippocampus E18 hippocampal neurons	Neuronal survival Synaptic and behavioral plasticity Dendritic growth	Kunert-Keil et al., 2006; Tai et al., 2008; Zhou et al., 2008; Boisseau et al., 2009; Du et al., 2010; Lin et al., 2013; Yao et al., 2013
**TRPC7**	Sprague-Dawley rats	Cholinergic interneurons, substantia nigra, subthalamic nucleus neurons	Downstream signaling to striatal mGluR1/5 receptors and NMDA-induced depolarization-activated inward current and firing	Zhu et al., 2005; Berg et al., 2007

## TRPC Canonical Channels in Parkinson’s Disease

Reports of the contribution of TRPC channels in PD are relatively new and we have not yet uncovered their definite roles in disease progression and maintenance. Also, few studies have attempted to link their expression and/or activation with the ongoing oxidative and nitrosative stresses that occur in PD.

TRPC1 is the most well investigated member of the canonical subfamily in PD. A study in SH-SY5Y cells demonstrated that TRPC1 protein expression becomes down-regulated in these cells following incubation with salsolinol (Bollimuntha et al., 2006), a neurotoxin endogenously found in the nigrostriatal cells and cerebrospinal fluid samples of patients with PD (Moser et al., 1995; Maruyama et al., 1996). Despite its low expression on the cell membrane, the TRPC1 protein was detected in the cytosol (Bollimuntha et al., 2006). This result suggests that salsolinol may cause TRPC1 translocation from the neuronal cell membrane to the cytoplasm.

Interestingly, the endogenous salsolinol derivative NMSAL was detected in the nigrostriatum and intraventricular fluid samples of patients with PD (Maruyama et al., 1996). NMSAL induces neuronal apoptosis via mitochondrial and caspase-3-dependent pathways (Akao et al., 1999; Maruyama et al., 2001; Arshad et al., 2014) and it is considered to be far more toxic to neurons than salsolinol (Maruyama et al., 1996). NMSAL exhibited similar effects to those of salsolinol in neuronal TRPC1 expression and localization (Arshad et al., 2014). All this evidence indicates a protective role for TRPC1 in PD.

Ca^2+^-induced ROS generation in cultured rat DA neurons treated with the neurotoxin 1-methyl-4-phenylpyridinium ion (MPP^+^) was also linked to TRPC1 (Chen et al., 2013). Another study by Selvaraj et al. (2009) showed that 1-methyl- 4-phenyl-1, 2,3,6-tetrahyrdro-pyridine (MPTP), a compound known to cause PD in mice by inducing mitochondrial dysfunction and neuronal apoptosis, reduces the expression of TRPC1 in the SNpc. A similar result was observed in PC12 cells incubated with MPP^+^. The same study also found that TRPC1 over-expression increases the survival of PC12 cells incubated with MPP^+^ by preserving mitochondrial membrane potential and regulating the expression of the anti-apoptotic genes Bcl2 and Bcl-xl (Selvaraj et al., 2009). Of note, the authors highlighted in their study that TRPC1 over-expression only partly restores mitochondrial membrane potential and neuronal survival.

The contribution of other TRPCs to PD has also been investigated. Analysis of TRPC3 expression patterns revealed that the TRPC3 protein is increased in the SNpc following exposure to MPTP (Selvaraj et al., 2009). On the other hand, no alterations in TRPC3 levels were noted in DA neurons from PD patients (Sun et al., 2017). Of note, these controversial data on TRPC3 expression have been obtained in different experimental settings. Therefore, TRPC3’s role in PD cannot be overruled. Also, it is possible that other TRPC channels and their complexes may contribute to changes in neuronal survival in PD.

In this context, it is important to highlight the complexes formed by TRPC1 with TRPC5. Although no studies have yet investigated these complexes in PD, they have been pointed as mediators of other neurodegenerative diseases such as Huntington’s. In a recent report, it was demonstrated that intracellular oxidized glutathione activates TRPC5 in striatal cells of Huntington’s disease (Q111 cells). The same study showed that upon oxidative stress, TRPC5-mediated Ca^2+^ influx leads to increased cytosolic Ca^2+^ levels and activation of the calpain-caspase pathway, leading to apoptosis of striatal neurons (Hong et al., 2015). In parallel, as observed for PD, TRPC1 protein and mRNA expression is down-regulated in Huntington’s striatal cells favoring the formation of TRPC5 heterotetramers in these cells (Hong et al., 2015). These results reinforce the protective role of TRPC1 in neurodegenerative diseases and shed light on the deleterious importance of TRPC5 in neuronal survival.

From the best of our knowledge, no studies have yet investigated the association between TRPC channels and RNI in PD, highlighting the need for further studies to fill this gap of information.

## Future Perspectives

Herein, we presented evidence and discussed the importance of TRPC channels in the recognition and regulation of oxidative and nitrosative stress responses, as well as their contributions to PD. The recent advances in the field of TRPC channels, in particular the protective functions of TRPC1 and the deleterious role of TRPC5 in PD, highlight their importance as pharmacological targets in treating neurodegenerative diseases. Considering the ability of TRPC channels to assemble as homo- and heterotetramers with channels of the same subfamily and also as members of other subfamilies of TRPs, and the lack of antagonists and agonists capable of selectively differentiating the individual actions of each one of these channels, their targeting of PD may become a difficult task. Therefore, efforts need to be made in order to develop effective and more selective pharmacological tools to investigate TRPC channels. This will be an essential step to achieve a broader knowledge of the pathophysiological roles of their different assembly modes and establish their definite importance in PD.

## Author Contributions

DM-F, NO, LS, and EF contributed to the conception and design and drafted and critically revised the manuscript. All authors gave final approval and agreed to be accountable for all aspects of the work.

## Conflict of Interest

The authors declare that the research was conducted in the absence of any commercial or financial relationships that could be construed as a potential conflict of interest.
